# Oropharyngeal Tularemia Outbreak Associated with Drinking Contaminated Tap Water, Turkey, July–September 2013

**DOI:** 10.3201/eid2112.142032

**Published:** 2015-12

**Authors:** Dilber Aktas, Bekir Celebi, Mehmet Emirhan Isik, Celal Tutus, Huseyin Ozturk, Fehminaz Temel, Mecit Kizilaslan, Bao-Ping Zhu

**Affiliations:** Public Health Institution of Turkey, Ankara, Turkey (D. Aktas, B. Celebi, C. Tutus, H. Ozturk, F. Temel);; Bayburt Public Hospital, Bayburt, Turkey (M.E. Isik);; Bayburt Provincial Public Health Directorate, Bayburt (M. Kizilaslan);; World Health Organization, European Regional Office, Turkey Country Office, Ankara (B.-P. Zhu)

**Keywords:** Tularemia, oropharyngeal tularemia, outbreak, water, tap water, contamination, cohort studies, Turkey, exposure period, epizootic disease, intracellular coccobacilli, Francisella tularensis subspecies holarctica, transmission, source, bacteria

## Abstract

In 2013, an oropharyngeal tularemia outbreak in Turkey affected 55 persons. Drinking tap water during the likely exposure period was significantly associated with illness (attack rate 27% vs. 11% among non–tap water drinkers). Findings showed the tap water source had been contaminated by surface water, and the chlorination device malfunctioned.

Tularemia, a severe epizootic disease caused by the gram-negative, intracellular coccobacillus *Francisella tularensis* (*1*,*2*), has 5 clinical forms: glandular/ulceroglandular, oculoglandular, pneumonic, typhoidal, and oropharyngeal (*2*). Oropharyngeal tularemia is caused by ingesting water or food contaminated with *F. tularensis*; the incubation period ranges from 1 to 14 days (*2*,*3*). Symptoms include sore throat, mouth ulcers, tonsillitis, and swollen lymph nodes in the neck.

Tularemia was first reported in Turkey in 1936. Subsequently, small outbreaks and sporadic cases have been reported, most of which were thought to be waterborne (*4*). In summer 2013, an outbreak of oropharyngeal tularemia occurred in a village in northeastern Turkey. We investigated the outbreak to identify the source of infection and mode of transmission. 

## The Study

On August 19, 2013, two persons from Sancaktepe Village, Turkey, sought care for influenza-like symptoms, tonsillitis, and swollen neck lymph nodes. Both patients tested positive for *F. tularensis* by blood microagglutination test. Clinicians treating the patients did not perform lymph node biopsies or conduct PCR testing of blood specimens to identify *F. tularensis*. Over the following weeks, dozens more patients were identified from the same village.

We conducted an investigation to identify potential exposures leading to *F. tularensis* infection (of any clinical form) among Sancaktepe Village residents. We defined a suspected case as onset of >1 specific symptoms (swollen lymph nodes in the neck or periauricular areas, sore throat, or swelling or redness of eyes) or >2 nonspecific symptoms (fever, chills, myalgia, or headache) during July 1–August 1, 2013. A probable case was onset of swollen lymph nodes plus sore throat or fever. A confirmed case was a suspected or probable case with a positive serologic test result.

We used the microagglutination test to detect *F. tularensis*–specific antibodies in patients’ blood; a titer >1:160 was the cut-off (*2*). Because of inadequate laboratory capacity to handle heavily polluted water, we used culture, but not PCR, to identify *F. tularensis* in implicated environmental samples.

Of 350 Sancaktepe Village residents, we excluded 46 who were absent during Ramadan 2013, the likely exposure period (explained in the next paragraph). From the remaining 304 residents, we identified 122 suspected case-patients, of whom 94 underwent blood microagglutination testing; 39 were positive (titers 1:160–1:2,560) for *F. tularensis*. No patient had a 4-fold rise in antibody titers between acute and convalescent phases of illness. On the basis of symptoms, we identified 16 additional probable cases among suspected case-patients who were not tested (7/13) or who had a negative microagglutination test result (9/24). The 55 confirmed or probable cases/case-patients are henceforth referred to as cases/case-patients.

The outbreak began on July 9, peaked in late July, and ended in early September 2013. Of the 304 residents, 55 (18%) were infected. The epidemic curve indicated a continuous common-source exposure and a likely exposure period that roughly coincided with Ramadan 2013 (July 8–August 7) (Figure). Cases occurred in all age groups (Technical Appendix Table 1) and village-wide. The attack rate did not differ significantly by sex: 18 (13%) of 137 male residents and 37 (22%) of 167 female residents were infected (relative risk [RR] 1.6, 95% CI 0.93–2.6). Clinical signs and symptoms included influenza-like symptoms and swollen lymph nodes in the neck or preauricular regions (Technical Appendix Table 2).

**Figure:**
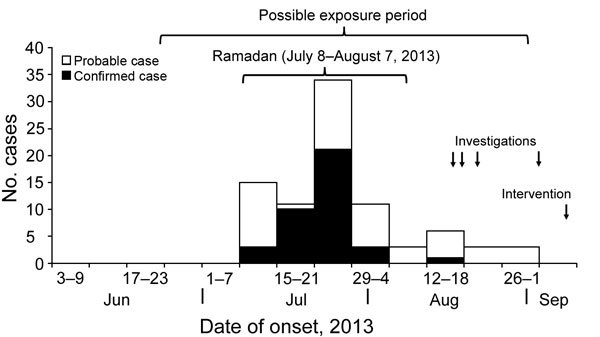
Epidemic curve indicating a continuous common-source exposure leading to an outbreak of oropharyngeal tularemia, Sancaktepe Village, Turkey, July–September 2013.

We hypothesized that the outbreak was caused by waterborne bacteria for 3 reasons: case-patients predominantly had oropharangeal symptoms; case-patients’ age and geographic distributions suggested a ubiquitous exposure; and villagers reported that the tap water had a dead-animal smell during Ramadan. A retrospective cohort study (excluding 46 persons with sore throat or swollen lymph nodes but not meeting definitions for probable or confirmed case-patients) showed that illness developed in 27% (46/173) of persons who drank tap water versus 11% (9/85) of persons who did not (RR 2.5, 95% CI 1.3–4.9). Other types of water were not associated with illness (Table). Sensitivity analyses showed slightly stronger associations between drinking tap water and illness when only confirmed cases (RR 3.4, 95% CI 1.4–8.4) or cases with onset during the week of July 22 (RR 3.0, 95% CI 1.1–8.4) were included.

**Table:** Risk for acquiring oropharyngeal tularemia among persons who drank water from different sources, Sancaktepe Village, Turkey, July–August 2013*

Source of water consumed	No. cases/total no. exposed (%)	No. cases/total no. not exposed (%)	Relative risk (95% CI)
Tap	46/173 (27)	9/85 (11)	2.5 (1.3–4.9)
Well	2/8 (25)	53/250 (21)	1.2 (0.35–4.00)
Underground spring	25/136 (18)	30/122 (25)	0.75 (0.47–1.2)
Bottled	5/31 (16)	50/227 (22)	0.73 (0.32–1.70)
Other	2/8 (25)	53/250 (21)	1.20 (0.35–4.00)

*The outbreak was associated with Ramadan, which occurred during July 8–August 7, 2013.

We asked villagers whether they had engaged in game hunting or eaten game meat around Ramadan; no villagers had such exposures. In addition, according to the village administrator, no large, village-wide gathering had occurred around Ramadan. Inspection of the village’s main water storage tank revealed that the solar-powered chlorination device had malfunctioned. Water collected on August 22 had a chlorine level of 0 and elevated levels of total coliform (60 colony-forming units [CFUs]) and *Escherichia coli* (1 CFU). The main water storage tank was supplied by 2 water collection sites, A and B. Water from site A had unremarkable findings and low turbidity. Collection site B had 3 sources of water, 1 of which was surface water. A water sample from site B had high turbidity and contained a visible insect. Rodent activities, but not dead animals, were evident near the surface water ditch. Meteorologic data showed a lack of precipitation in this area for months. Water samples collected from site B on August 22 had high levels of total coliform (>100 CFU) and *E. coli* (50 CFU) (Technical Appendix Figure). Culture of 2 water samples collected on August 28 and September 4, respectively, did not yield *F. tularensis*.

More than 300 wild and domestic animals worldwide have been found to be naturally infected with *F. tularensis* (*1*). *F. tularensis* subsp. *holarctica*, the only known disease-causing subspecies in Eurasia (*5*), is associated with water-associated rodents (e.g., beavers, muskrats). Humans can be infected with this subspecies by drinking contaminated water; having contact with contaminated streams, lakes, or rivers; having direct contact with contaminated objects (*1*,*2*); or eating uncooked contaminated food (*6*).

Tularemia surveillance in Turkey reported 4,827 tularemia cases nationwide during 2005–2011; contaminated water was presumed to have caused most cases, especially in rural areas (*4*). *F. tularensis* subsp. *holarctica* has been isolated from drinking water sources in places where tularemia outbreaks occurred (*7*). The bacteria presumably came from dead animals; a single infected water animal (e.g., vole, lemming, or mouse) can contaminate up to 500,000 L of water (*1*), and *F. tularensis* can survive in untreated water for months (*2*). Free available chlorine residual concentrations routinely maintained in tap water systems can reduce *F. tularensis* by 4 log_10_ in 2 hours (*8*). However, the malfunction of the chlorination device at Sancaktepe Village’s main water storage tank enabled survival of the bacteria.

Our study had several limitations. *F. tularensis* was not isolated from water. *Francisella* species are fastidious and slow-growing and can be easily overwhelmed by competing organisms in environmental samples during culture (*9*). In addition, water samples were collected during late August–early September; by that time, the bacteria might have been cleared from the water. We spotted rodent activities, but no dead animals, near the implicated water source. The imperfect case definitions and potential subclinical infections in asymptomatic villagers might have led to misclassification, which tends to bias the association toward null; in other words, the observed association would have been stronger had there been no such bias, as evidenced by the sensitivity analysis that used laboratory-confirmed cases only.

## Conclusions

This tularemia outbreak in northeastern Turkey was associated with drinking contaminated tap water. At our recommendation, the village administrator cut off the surface water source, repaired the chlorination device, and started checking chlorine levels regularly. No new outbreaks have subsequently occurred.

Technical Appendix Age distribution and clinical and signs and symptoms of probable and confirmed case-patients and findings of environmental investigations during a tularemia outbreak, Sancaktepe Village, Turkey, July–August 2013.
