# Supplementary material for: Oropharyngeal Tularemia Outbreak Associated with Drinking Contaminated Tap Water, Turkey, July–September 2013
Source: Emerg Infect Dis. 2015 Dec;21(12):2194–6. doi: 10.3201/eid2112.142032 (PMC4672448; doi:10.3201/eid2112.142032)
Supplement: Technical Appendix — Age distribution and clinical and signs and symptoms of probable and confirmed case-patients and findings of environmental investigations during a tularemia outbreak, Sancaktepe Village, Turkey, July–August 2013. [file 14-2032-Techapp-s1.pdf]

Article DOI: http://dx.doi.org/10.3201/eid2112.142032

Oropharyngeal Tularemia Outbreak Associated with Drinking Contaminated Tap Water, Turkey, July–September 2013

Technical Appendix

Technical Appendix Table 1. Age distribution of probable and confirmed case-patients during a tularemia outbreak, Sancaktepe Village, Turkey, July–August 2013

Age group, y	No. cases /no. persons total (%)
1–10	9/36 (25)
11–20	7/50 (14)
21–40	12/73 (16)
41–60	15/82 (18)
61–90	12/63 (19)
All	55/304 (18)

Technical Appendix Table 2. Clinical signs and symptoms of probable and confirmed case-patients during a tularemia outbreak, Sancaktepe Village, Turkey, July–August 2013

Signs and symptoms	No. (%) cases, N = 55
Chills	46 (84)
Sore throat	45 (82)
Fever	40 (73)
Headache	40 (73)
Myalgia	32 (58)
Swollen lymph nodes in neck or periauricular regions	32 (58)
Vomiting and/or diarrhea	20 (36)
Swelling or redness of eyes*	14 (26)

*All of these case-patients also had sore throat or swollen lymph nodes in neck or periauricular regions.

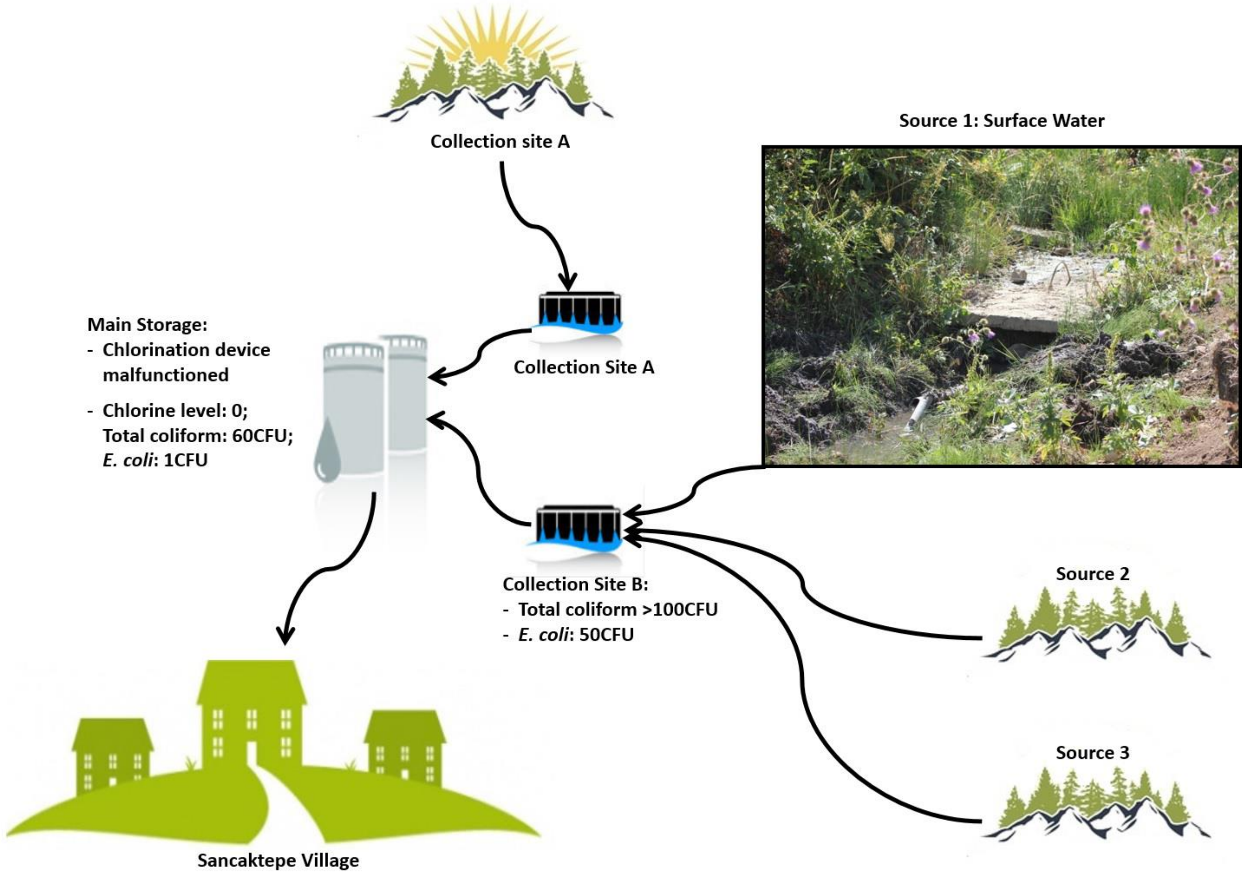

Technical Appendix Figure. Tap water system and findings of environmental investigations during a tularemia outbreak, Sancaktepe Village, Turkey, July–August 2013.
